# A 3D Printed Device for Easy and Reliable Quantification of Fungal Chemotropic Growth

**DOI:** 10.3389/fmicb.2020.584525

**Published:** 2020-11-03

**Authors:** Carolin Schunke, Stefanie Pöggeler, Daniela Elisabeth Nordzieke

**Affiliations:** Department of Genetics of Eukaryotic Microorganisms, Institute of Microbiology and Genetics, Georg August University Göttingen, Göttingen, Germany

**Keywords:** chemotropism, 3D printed device, glucose, *Colletotrichum graminicola*, filamentous fungi

## Abstract

Chemical gradients are surrounding living organisms in all habitats of life. Microorganisms, plants and animals have developed specific mechanisms to sense such gradients. Upon perception, chemical gradients can be categorized either as favorable, like nutrients or hormones, or as disadvantageous, resulting in a clear orientation toward the gradient and avoiding strategies, respectively. Being sessile organisms, fungi use chemical gradients for their orientation in the environment. Integration of this data enables them to successfully explore nutrient sources, identify probable plant or animal hosts, and to communicate during sexual reproduction or early colony development. We have developed a 3D printed device allowing a highly standardized, rapid and low-cost investigation of chemotropic growth processes in fungi. Since the 3D printed device is placed on a microscope slide, detailed microscopic investigations and documentation of the chemotropic process is possible. Using this device, we provide evidence that germlings derived from oval conidia of the hemibiotrophic plant pathogen *Colletotrichum graminicola* can sense gradients of glucose and reorient their growth toward the nutrient source. We describe in detail the method establishment, probable pitfalls, and provide the original program files for 3D printing to enable broad application of the 3D device in basic, agricultural, medical, and applied fungal science.

## Introduction

Polar tip growth of long tubular hyphae is a fundamental characteristic of filamentous fungi (Momany, 2002). Since fungi are sessile organisms, they have to adapt dynamically to changes of the environment, re-evaluate the current growth direction and probably re-orient their growth. Thereby, environmental stimuli like light (phototropism), contact (thigmotropism), electrical currents (galvanotropism), and chemicals (chemotropism) can provoke a re-direction of growth (Corrochano and Cerdá-Olmedo, 1991; Brand and Gow, 2009; Turrà et al., 2016). The nature of chemical cues ranges from nutrients sources, host-derived signals to compounds governing fungal communication, such as pheromones. Sensing of these compounds can result in both positive and negative growth responses (Massee, 1905; Turrà et al., 2016). Intriguingly, fungi employ a prioritization of signals perceived, allowing them to discriminate between different options to identify the optimal path (Turrà et al., 2016). Despite the early identification of chemotropic processes in fungi and their role for interorganismic interaction during pathogenicity and symbiosis (de Bary, 1884), knowledge about signals and molecular factors involved in signal sensing and growth re-direction is still limited. In parts, this is due to a lack of easy methodical approaches to gain reproducible results. A method allowing the detailed investigation of chemotropic growth responses in substantial fungal processes such as mating, fusion of fungal germlings during early colony development, pathogenic as well as mutualistic interactions with plant and mammalian hosts would be highly valuable for the fungal research community (Brand and Gow, 2009; Takeshita, 2016).

In our lab, we work with the hemibiotrophic plant pathogen *Colletotrichum graminicola* (Ces.) G.W. Wils., originally provided by R. L. Nicholson, Purdue University, IN. This fungus belongs to the worldwide-distributed *Colletotrichum* species complex (O’Connell et al., 2012), causing anthracnose on leaves and stalks on its host *Zea mays* and also infects the roots of its host (Bergstrom and Nicholson, 1999; Sukno et al., 2008). Due the ability to infect several plant parts and its high epidemic spreading potential, this pathogen is estimated to cause crop losses up to 1 billion dollars/year (United States) corresponding to 40% per field (Perfect et al., 1999; Frey et al., 2011). *C. graminicola* generates two types of infectious asexual spores, falcate and oval shaped conidia (Nishihara, 1975; Panaccione et al., 1989), having distinct roles and following particular strategies during the infection cycle (Nordzieke et al., 2019b). As we recently reported, oval conidia develop a germling network by the formation of conidial anastomosis tubes (CATs) on maize leaves, which might contribute to their pathogenic potential (Nordzieke et al., 2019b). In contrast, falcate conidia germinate on short germ tubes on maize leaves followed by rapid development of appressoria for plant penetration (Mims and Vaillancourt, 2002). Since root infection as well as CAT formation requires chemotropic sensing of host roots or fungal germlings, respectively, we sought for an easy, low cost method allowing reliable examination of chemotropic growth responses in *C. graminicola*.

In the last years, microfluidic devices were used in several studies to investigate the process of pheromone gradient sensing and the formation of mating projections (shmoos) of unicellular yeast (Paliwal et al., 2007; Moore et al., 2008). In this setup, changing gradient application due to pump rearrangement makes it possible to investigate re-orientation of shmoos (Brett et al., 2012). For filamentous fungi, several microfluidic devices were developed to investigate polar growth, germination, sporulation, nutrient distribution, response upon fungivore attack, and circadian rhythms. They also serve as a platform for high-throughput screening for secreted enzyme activities (Lee et al., 2013; Geng et al., 2015; Marshall et al., 2017; Schmieder et al., 2019). However, these approaches fail to depict growth re-orientation processes. To cope with this methodical gap, Turrà et al. (2015) developed a method termed “chemotropic assay” to investigate growth re-orientation processes of *Fusarium oxysporum* f. sp. *Lycopersici* germlings, a pathogen of tomato roots (Turrà et al., 2015). In contrast to most microfluidic devices, this method is based on slow gradient establishment in water agar. When the signal gradient reaches the fungus, most of the embedded *F. oxysporum* conidia have germinated and established a general growth direction. Sensing of the gradient signal results in a re-direction of their polar axis. The re-orientation of the germ tube tip can be determined by microscopic investigations of the projection angle in analogy to investigations in yeast (Brett et al., 2012; Turrà et al., 2015). However, to learn the handling of the method is rather time consuming and several drawbacks hinder the employment. First, exact parallel wells for signal application have to be drawn by hand to enable uniform gradient formation. Further, fixing of the reaction chamber, a dish, under a common microscope is difficult and hinders the readout procedure. Third, since dishes are not adapted for microscopic approaches, the investigation of fluorescent-labeled proteins during the chemotropic process is not possible.

We describe here the design of a 3D printed device consisting of a frame and combs fitting on custom microscope slides, overcoming most of the drawbacks present in the original chemotropic assay. For the validation process, we use oval conidia of the plant pathogen *C. graminicola*. We provide evidence that germlings derived from these conidia are able to re-direct their growth to gradients of 50 mM glucose in a timeframe of 6 h using the original method of Turrà et al. (2015) as a control. Together, the here presented method allows easy and low-cost elucidation of fungal chemotropic mechanisms and can push forward research in basic and applied science.

## Materials and Equipment

### Implementation of the 3D Printed Device

- Device design: Inventor 2019 (Autodesk, Mill Valley, CA, United States)
- Ultimaker 3 printer (FDM) equipped with the Cura software (Ultimaker, Geldermalsen, Netherlands)
- Material for printing: acrylonitrile butadiene styrene polymer

### Fungal Culture

- Complete medium sucrose (CMS): glucose 10 g/L, yeast extract 1 g/L, peptone 1 g/L, solution A [Ca(NO_3_)_2_ 100 g/L] 10 ml/L, solution B (KH_2_PO_4_ 20 g/L, MgSO_4_ 25 g/L, NaCl 5.4 g/L) 100 ml/L, sucrose 171.15 g/L
- 250 ml Erlenmeyer not baffled flasks with cotton plugs, sterile
- Minitron incubator (INFORS HAT, Bottmingen, Germany)
- Plastic funnel
- Miracloth (EMD Millipore Corp., Billerica, MA, United States)
- Centrifuge 5810 R (Eppendorf AG, Hamburg, Germany); tubes 50 ml
- Falcate conidia of *Colletotrichum graminicola* (Ces.) G.W. Wilson (teleomorph Glomerella graminicola D. J. Politis)
- Hemacytometer acc. to Neubauer Improved

### Chemotropic Growth Assay

- Melted basic agar (Agarose (1% w/v), Serva Agar (1% w/v) in water)
- Melted top agar (Agarose (0.25% w/v), Serva Agar (0.25% w/v) in water)
- Microscope slides, cover slips
- 3D printed device
- Blue pen, set square
- Oval conidia of *C. graminicola* wildtype, c = 5^∗^10^5^/ml in water

## Methods

Directed growth to chemical gradients is fundamental to fungal lifestyle. However, current approaches to study the corresponding processes are limited or error prone. We have developed an assay to allow easy and standardized investigation of chemotropic responses using a new-engineered 3D printed device.

### Establishment

#### 3D Printed Device

**FIGURE 1:**
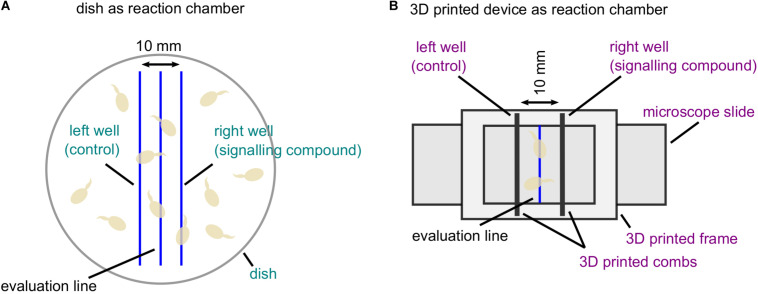
Comparison of two different reaction chambers for the analysis of directed fungal growth. **(A)** Classical setup of the chemotropic assay based on a dish as reaction chamber (Turrà et al., 2015). A dish is filled with water agar mixed with fungal conidia (light brown). Along hand-drawn parallel lines, two wells are cut serving the application of the signaling compound and the solvent control. Along the evaluation line, the orientation of germ tube tips is evaluated. Error-prone methodical parts are indicated in turquoise. **(B)** Chemotropic assay using the new 3D printed device consisting of a frame and two combs. The 3D printed frame is fixed on a microscope slide coated with water agar providing a hand-drawn evaluation line. Two 3D printed combs are placed in provided slots with a distance of 10 mm to create reproducible wells. Fungal conidia (light brown) are applied on water agar topping the evaluation line. Improvements of the methodical setup are marked in violet.

The basis for the development of the 3D printed device was the chemotropic assay for the analysis of directed growth responses of *F. oxysporum* published previously (Turrà et al., 2015). Due to the usage of a dish as reaction chamber in this assay, several error sources may influence the reliability of the obtained results (Figure 1A). The aim of the new device is to simplify the methodical setup and readout procedure. In short, the 3D printed frame is designed to fit on a microscope slide, which can be directly used for the quantification of fungal growth by microscopy. To allow constant signal gradient formation, we also designed combs by 3D printing. These fit in specific slots in the 3D printed frame, allowing the generation of wells in a distance of 10 mm to each other (Figure 1B and Supplementary File 1). In both methods, the strict vertical evaluation line is drawn by hand centering the two wells, serving as optical line for the examination of chemotropic growth. We have equipped the bottom side of the 3D printed device with two notches in the center between the wells to ensure exact positioning of this optical line (Table 1). In principal, water agar volumes from 1.5 to 4 ml fit into the 3D printed device, which allow for different volumes of signal application. Here, we are using 2.5 ml of water agar and 40 μl of chemoattractant as standard volumes.

**TABLE 1:** Improvement of the chemotropic assay by the newly designed 3D printed device.

**Dish as reaction chamber – Probable error sources**	**3D printed device as reaction chamber -Standardized procedure**
Exact parallel wells for signal application must be drawn by hand to allow uniformity and reproducibility of gradient formation	3D printed combs and comb positioning slots allow the exact reproduction of uniform signal gradients
Evaluation line centering the two wells is drawn by hand	Positioning notches allow exact centering of evaluation line in between the two wells
Microscope tables are not optimized in holding round plates making the exact positioning of the evaluation line difficult	3D printed frame fits on standard microscope slides, allowing the exact and easy positioning of the evaluation line
Dishes are not optimized for light microscopy thus complicating documentation	The microscope slide as basis simplifies documentation and allows simultaneously usage of fluorescent markers and labeled proteins
	

#### Choice of Chemoattractant Signal

Directed growth toward chemical stimuli is fundamental to filamentous fungi. However, the nature of signals provoking directed growth responses differs between fungal species due to their distinct lifestyles (Massee, 1905). It is reasonable that besides these species-specific signals, a set of “common” molecules exists with the general ability to attract filamentous fungi. One of the oldest known chemoattractant molecules is glucose, described as being able to attract fungi like *F. oxysporum* and *Botrytis vulgaris* (Miyoshi, 1894; Fulton, 1906; Turrà et al., 2015). For *F. oxysporum* it was reported that 1% of glucose (55.6 mM) has a high chemoattracting potential (Turrà et al., 2015). Anticipating that *C. graminicola* might be able to sense similar concentrations, we used 50 mM of glucose as chemoattractant throughout this study.

#### Fungal Growth Conditions

**FIGURE 2:**
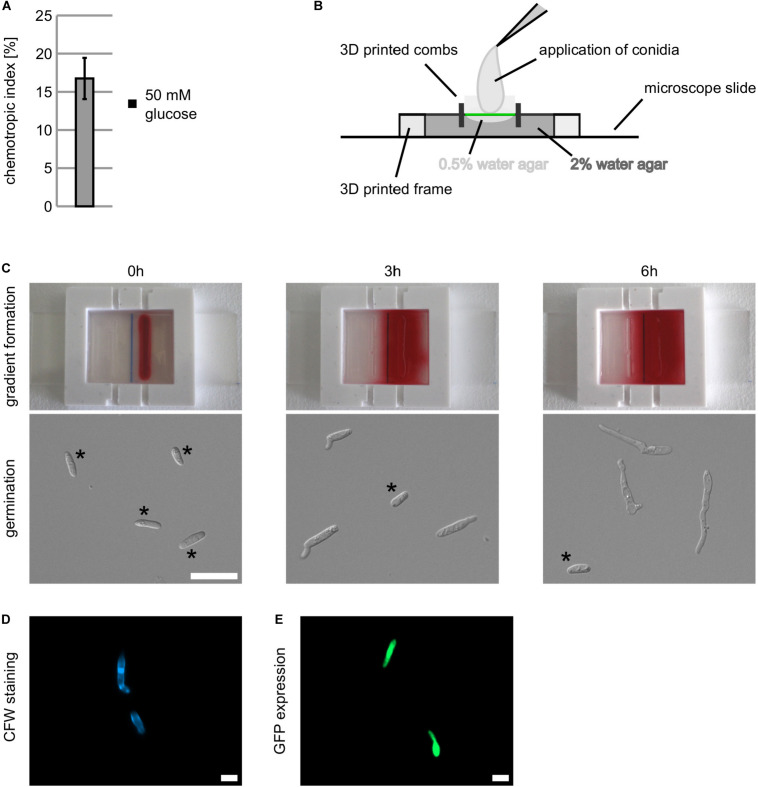
Establishment of an operation mode to measure chemotropic growth responses using the new generated 3D printed device. **(A)** Chemotropic growth of *C. graminicola* oval conidia to a gradient of 50 mM of glucose quantified with the chemotropic plate assay, error bars represent SD calculated from *n* = 5 experiments as indicated. **(B)** Arrangement of 3D printed frame and 3D printed combs on an microscopic slide. 2% of water agar [1% (w/v) agarose, 1% (w/v) agar (Serva)] is fixing the device on the microscope slide (dark gray). Due to surface tension, a concave surface forms in between the created wells. To even the surface (green line), 0.5% of water agar [0.25% (w/v) agarose, 0.25% (w/v) agar (Serva)] are applied (light gray). **(C)** Depiction of gradient buildup over time by spread of Ponceau stain and respective germination of *C. graminicola* oval conidia. Conidia not germinated are indicated*. **(D,E)** Visualization of *C. graminicola* germlings stained with 1:1 diluted calcofluor white (CFW, **D**) and expressing cytosolic GFP **(E)** after incubation in the 3D printed device for 6 h, size bar = 10 μm.

*C. graminicola* generates two distinct asexual spores, falcate and oval conidia, differing in morphology, germination behavior, and maize infection strategies (Panaccione et al., 1989; Nordzieke et al., 2019b). Since *C. graminicola* oval conidia are morphologically similar to microconidia of *F. oxysporum* used in former studies (Turrà et al., 2015; Nordzieke et al., 2019a; Vitale et al., 2019), we adapted fungal-specific conditions for oval conidia and optimized incubation time, water agar composition and spore concentration alike as the spore application procedure. Here, we obtained best results applying 40 μl of oval conidia (c = 5^∗^10^5^/ml) on water agar (0.25% (w/v) agarose mixed with 0.25% (w/v) agar (Serva)) layered on the evaluation line instead of spreading the spores into the water agar (Figure 1B). After 6 h of incubation, we determined a chemotropic response of 16.7% (± = 2.7%) for *C. graminicola* oval conidia to a gradient of 50 mM glucose (Figure 2A). For *C. graminicola*, we recommend not to extend the incubation time to more than 8 h.

#### Assembling and Gradient Formation

In a first step, we had to identify the best matrix for fungal growth and gradient establishment. We put the 3D printed frame on a microscope slide and inserted the combs in the designated positions to create wells for the application of the chemoattractant signal. Then we filled 2.5 ml of 2% water agar [1% (w/v) agarose 1% (w/v) agar (Serva)] inside the device to fix it to the microscopic slide (Figure 2B). After solidification, we observed that the surface of the agar between the combs was concave, hampering the application of a cover glass for microscopy (Figure 2B, dark gray). To provide an even surface, we filled 0.5% water agar [0.25% (w/v) agarose mixed with 0.25% (w/v) agar (Serva)] upon the solidified water agar in between the combs, resulting in an even surface for the application of oval conidia and a cover glass (Figure 2B, light gray). After removal of the combs, we applied 40 μl of an aqueous Ponceau red solution in the right well and monitored time-dependent gradient formation (Figure 2C and Supplementary File1). After 6 h, a robust and uniform gradient is established, matching optimal time for germination of *C. graminicola* oval conidia on water agar (Figure 2C). To investigate chemotropic growth in greater depth, our experimental setup allows the visualization of specific cellular structures by fluorescence microscopy. As examples, we show germlings either stained with an aqueous 1:1 calcofluor white (CFW) solution (Figure 2D) or expressing a green fluorescence protein (GFP; Weihmann et al., 2016; Figure 2E).

#### Evaluation of Directed Growth Patterns

**FIGURE 3:**
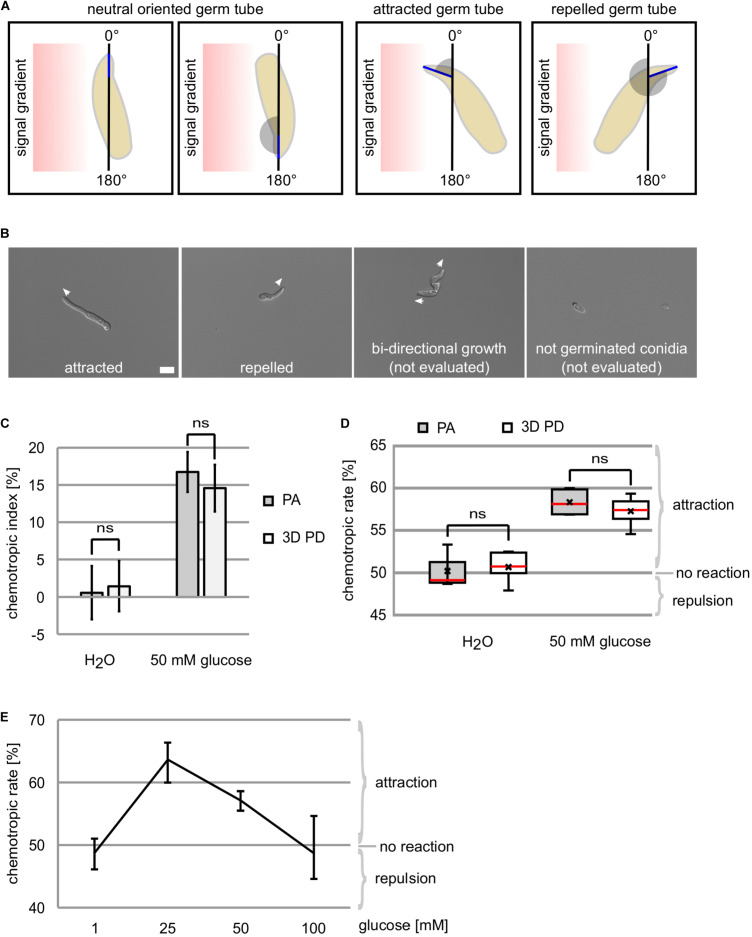
Chemotropic growth of *C. graminicola* oval conidia. **(A)** Determination of the hyphal tip projection angle (blue) allows the evaluation of germ tube growth directions as attracted (1°–179°), repelled (181°–359°), or neutral (0°/360° and 180°). **(B)** Evaluation of germ tube growth direction to a gradient of 50 mM glucose after incubation for 6 h, size bar = 10 μm. **(C–E)** Growth re-direction of germlings derived from *C. gramincola* oval conidia to glucose gradients is quantified using the original plate assay (PA) and the new developed 3D printed device (3D PD) after 6h of incubation at 23°C, *n* ≥ 4, ns = not significant (*p* > 0.05). **(C)** Depiction of results as deviation from the expected attraction potential of the negative control (water), termed chemotropic index. For each replicate, a minimum of 70 germlings were evaluated. **(D)** Data are provided as chemotropic rate, giving the ratio of attracted to total germlings. For each replicate, a minimum of 40 germlings were evaluated. Meridian is indicated in red, average by x. **(E)** Dose-response curve of germlings to glucose concentrations as indicated quantified using the 3D printed device. For each replicate, a minimum of 40 germlings were evaluated.

For the evaluation whether or not a germ tube tip directs to an applied chemoattractant, the hyphal tip projection angle is used along an imaginary vertical line (Figure 3A). However, besides clearly attracted or repelled germ tube tips, the possibility exists that a germling shows bi-directional growth or that a conidium is not germinated during the incubation time. Since in both latter cases no clear growth direction can be determined, these are not included into our evaluation (Figure 3B).

When the growth direction of all germ tube tips is examined, there are three possible outcomes how the total of germlings react to the chemoattractant applied:

- **Equal distribution** between fungal tips growing to the chemoattractant signal and the negative control (positive: negative/50%: 50%). This distribution is expected when the tested **compound has not the ability to change the growth direction** of the fungus.
- **More fungal tube tips** are pointing **toward the chemoattractant signal** compared to the control (positive: negative/ > 50%: <50%). This result corresponds with an **attracting potential** of the tested compound.
- **Less fungal tube tips** are pointing **toward the chemoattractant signal** compared to the control (positive: negative/ < 50%: > 50%). This result corresponds with a **repelling potential** of the tested compound.

So far, the quantified chemotropic growth response is depicted using the chemotropic index. This index bases on the deviation of the obtained results from the equal distribution as the expected situation (Turrà et al., 2015):

$$\mathrm{chemotropic}\mathrm{index}\left[\%\right]=\left(\left\{\left(\frac{\mathrm{number}\,\mathrm{of}\,\mathrm{attracted}\,\mathrm{fungal}\,\mathrm{tips}}{\mathrm{sum}\,\mathrm{of}\,\mathrm{all}\,\mathrm{fungal}\,\mathrm{tips}}\right)\times 100\right\}-50\right)\times 2$$

As an alternative, we propose to display the quantified chemotropic growth as box plot giving the direct rates from attracted and repelled hyphal tips:

$$\mathrm{chemotropic}\mathrm{rate}\left[\%\right]=\left(\frac{\mathrm{number}\,\mathrm{of}\,\mathrm{attracted}\,\mathrm{fungal}\,\mathrm{tips}}{\mathrm{sum}\,\mathrm{of}\,\mathrm{all}\,\mathrm{fungal}\,\mathrm{tips}}\right)\times 100$$

In Figures 3C,D, the representation of the chemotropic index and the chemotropic rate are compared to each other.

#### Probable Pitfalls

For the original assay alike as the here described one it is vital to take into account the optical path of the used microscope to allow correct classification of germlings as “attracted” or “repelled.” We recommend a maximum imaging time of 20 min per experiment.

### Stepwise Procedures

#### Printing of 3D Device

3D printed frame and combs are designed using the Autodesk Inventor 2019 software. After export to.stl files (Supplementary Files 2, 3), both files are imported in the “Ultimaker Cura” program. Printing of an acrylonitrile butadiene styrene polymer was conducted with a “Ultimaker3” 3D printer using the FDM technology for layer-by-layer printing. General settings of the 3D printer are given in Table 2.

**TABLE 2:** Settings of 3D printer.

**Variables**	**Setting**	**Measure**
Quality	General layer thickness	0.15 mm
	Thickness of first layer	0.27 mm
	Line thickness	0.35 mm
	Line thickness of first layer	120%
Case	Section thickness	1.3 mm
	Number of section lines	4
	Thickness of down/top layer	1.2 mm
Filling	Filling factor	35%
	Filling pattern	Triangles
Material	Temperature	235°C
	Temperature of printing plate	105°C
Speed	Printing speed	60 mm/s
	Printing speed for first layer	20 mm/s

#### Preparation of the *C. graminicola* Inoculum

For the generation of *C. graminicola* oval conidia, 300 ml glass flasks are filled with 100 ml of complex medium with saccharose (CMS) and inoculated with 50 μl of falcate conidia obtained from a fresh plate culture [oat meal agar (OMA), 14–21 days] or a deep frozen glycerol stock (final concentration 50% glycerol) (Leach et al., 1982; Werner et al., 2007). Cultures are incubated at 23°C for 2 days in shaking conditions (80 rpm) followed by 5 days static incubation in the dark. After a total of 7 days, mycelia and liquid phase are separated by miracloth filtration. After two rounds of centrifugation (10 min, 7,000 rpm) and washing of the pellet with distilled water, oval conidia are quantified using a Neubauer counting chamber (Nordzieke et al., 2019b). Finally, spore concentration is adapted to c = 5^∗^10^5^/ml in distilled water.

#### Preparation of Chemotropic Assay

All following steps are performed at a clean bench. First, a blue vertical evaluation line is drawn on a microscope slide and its position adjusted with the help of the notches. Then the 3D printed frame is fixed on the slide with 2.5 ml 2% of water agar [1% (w/v) agarose 1% (w/v) agar (Serva)], allowing the application of 40 μl of chemoattractant chemical. The middle area framed by the 3D printed combs is repeatedly filled with 100 μl of 0.5% of water agar [0.25% (w/v) agarose mixed with 0.25% (w/v) agar (Serva)] until an even surface is created. After removal of the combs, 20 μl of conidial suspension (c = 5^∗^10^5^/ml) is equally applied on the evaluation line and dried for 2–5 min under the laminar flow. 40 μl of the chemoattractant compound (50 mM glucose) and the corresponding solvent control (water) are applied in the right and left well, respectively. The microscope slide is now placed into a square dish and incubated for 6 h at 23°C (Supplementary File 1). After incubation time, the 3D printed frame is removed, leaving the agar block with the germlings and the signal gradient on the glass slide. A cover slip is placed on top of the middle area and the slide is fixed on an upright microscope table. Using 10x magnification, the evaluation line is centered. Along this line, microscopic pictures of growing germlings are taken with the 40x objective (DIC).

#### Quantification of Directed Growth Events

By means of the microscopic pictures taken, the hyphal tip projection angle is determined in analogy to the yeast system along a virtual vertical line as 0° reference (Brett et al., 2012; Figure 3A). Evaluation of the angle allows to sort the germ tubes into the three categories: (1) attracted (1°–179°), 2) repelled (181°–359°), and (3) neutral (180° or 0°/360°) (Figure 3A). A minimum of 40 germlings are analyzed for each experiment. Neutral positioned germ tube tips are not included in the evaluation. When a germling grows with two germination tubes, directional growth is only recorded when both tips are sorted into the same category. Based on the obtained numbers, the chemotropic index or the chemotropic rate are calculated.

## Results

### Oval Conidia of *C. graminicola* Redirect Their Growth to Glucose Gradients

In this study, we aimed to develop an improved method for the analysis of chemotropic growth responses enabling the study of different chemotropic responses in *C. graminicola*. In a first step, we tested if germlings derived from oval conidia of this fungus are able to sense glucose gradients. Using the published method from Turrà et al. (2015) and the method we describe here, we provide evidence that *C. graminicola* germlings adapt growth direction in a time corridor of 6 h to 50 mM glucose giving a chemotropic index of 16.7% (± 2.7%) and 14.6% (± 3.2%), respectively (Figures 2A, 3). In control experiments using water as neutral compound, no directed growth was detectable (Figure 3). The results obtained by the application of the different methods are statistically not different (*p* > 0.05) showing that the 3D printed device can be used as alternative to the dish-based assay (Figure 3). To elucidate whether other concentrations of glucose are able to provoke chemotropic re-direction in *C. graminicola* germlings, we used the 3D printed device to test 1, 25 mM as well as 100 mM of glucose as chemoattractants. Consistent with previous observations of fungal chemoattraction (Segall, 1993; Turrà et al., 2015), we obtained a dose response curve in which 25 mM and 50 mM of glucose result in strong chemoattraction, whereas 1 and 100 mM did not provoke redirection of growth (Figure 3E).

## Discussion

In the last years, several microfluidic devices were designed for the small-scale investigation of growth responses of bacteria, tumor cell lines, yeast but also multicellular organisms by the application of fluids, which overflow the biological material (Moore et al., 2008; Brett et al., 2012; Choi et al., 2012; Yanagisawa and Higashiyama, 2018). Unicellular organisms can be investigated quite easily with these systems: Liquid chemical gradients are established and can be changed by the utilization of pumps applying solutions from source to sink. The position of single cells can be tracked by fluorescence labeling, allowing retracing the movement reaction in detail (Wheeler et al., 2003; Choi et al., 2012; Sai et al., 2016). This is especially interesting for the investigation of tumor migration, since here even the invasion of tissue by single tumor cells can be monitored by applying different fluorescence markers for each cell type (Sai et al., 2016). In yeast, microfluidic devices are used to monitor growth responses of mating projections (Brett et al., 2012). By the application of yeast strains expressing fluorescence-labeled proteins, even the screening for proteins involved in the chemotropic growth of smooths was reported (Yuan et al., 2016). However, the application of a stable gradient throughout the experiment is insufficient to identify cells deficient in shmoo growth adaptation. Coping with this problem, Brett et al. (2012) designed a microfluidic device allowing the dynamic change of applied pheromone gradients. As a result, re-direction of shmoo cells can be visualized (Brett et al., 2012). An example for multicellular tissue reorienting to chemical signals, are pollen tubes. These have to reach the plant ovary to apply the non-motile sperms for the fertilization process (Higashiyama and Yang, 2017). Recently, a microfluidic device was designed to investigate this process. In contrast to the other approaches discussed, the signaling gradient is not established by liquids, but by the application of a dissected ovary in a chamber separated by the pollen tubes. Cells, which aim to reach the ovary, have to grow toward a solid matrix of agar and fulfill two directional changes. Due to this dual direction change, the number of false results is minimized (Yanagisawa and Higashiyama, 2018). Similar as described for pollen tubes, fungi are prone to grow toward gradients established in solid matrices by polar elongation. However, fungi are not focused on a special signaling gradient as pollen tubes but are able to sense various chemical compounds and therefore an adequate method has to enable the application of different chemicals (Gooday, 1975; Jansson et al., 1988; Turrà et al., 2016). In contrast to pollen tubes, fungal cells are also constantly interacting with each other, resulting in the formation of cellular fusions of hyphae, germlings and conidia (Fleissner and Herzog, 2016). Consequently, cross signaling has to be avoided by a strictly controlled cell number and density to exclude mixing of growth response outputs throughout the experiment (Moore et al., 2008). An optimal stadium to allow such controlled conditions is the early growth stage of fungi consisting of spores and germlings (Turrà et al., 2015). The here developed method combines advantages of approaches previously reported. Similar to the chemotropic assay established by Turrà et al. (2015), solution of chemicals can be applied reaching the fungal germlings after a precise time of incubation. Further, the 3D printed device allows a standardization of the process that is comparable to a microfluidic device. The here presented method permits further the investigation of fluorescent-labeled proteins during chemotropic growth responses, enabling the usage of marker proteins and localization studies during fungal growth redirection.

*C. graminicola* is an ascomycetous fungus infecting several plant parts of its host *Zea mays*, including leaves and roots, but can also live on dead organic matter (Bergstrom and Nicholson, 1999; Sukno et al., 2008). Despite the formation of CATs during early colony development, *C. graminicola* needs to re-direct growth for successful plant infection for example during penetration peg emergence and, presumably, for root infection. For the first time our study provides evidence that germlings derived from oval conidia *C. graminicola* are able to sense applied glucose concentrations of 25 mM and 50 mM and to adapt their polar axis toward the signal (Figure 2A). Intriguingly, in the bell-shaped dose response curve low and high glucose concentrations do not provoke growth re-direction (Figure 3E). This is in line with previous reports from yeast and *F. oxysporum* about chemotropic growth to distinct pheromone and glucose concentrations, respectively (Segall, 1993; Turrà et al., 2015). Somewhat counterintuitive, the bell-shaped curve reveals the difference between a chemotropic response to glucose and a mere influence of the nutrient on general growth patterns. In the latter case, we would expect faster growth with augmenting glucose concentrations applied. For fungi, several receptors for the sensing of extracellular nutrients have been reported (Xue et al., 2008). The 7 transmembrane G-protein coupled receptor (GPCR) Gpr1 of *S. cerevisiae* requires about 20–30 mM of glucose for its half-maximal activation (EC50) (Versele et al., 2001). However, it is difficult to speculate if a homolog protein might be involved in chemotropic glucose sensing in *C. graminicola*. As often, the investigation of Gpr1 homologs in filamentous fungi did not reveal a homolog function in glucose sensing as reported for yeast (Miwa et al., 2004; Maidan et al., 2005). In *Neurospora crassa* and *Schizosaccharomyces pombe* two distinct GPCRs were identified as probable glucose sensing receptors, but both are not homologous to Gpr1 (Welton and Hoffman, 2000; Li and Borkovich, 2006). In *F. oxysporum*, the FMK1 MAPK module homologous to the *S. cerevisiae* pheromone pathway as well as the transcription factor STE12 were shown to be involved in sensing of glucose gradients (Turrà et al., 2015). Whether these factors play also a role for chemotropic glucose sensing and which other compounds might induce growth re-direction in *C. graminicola* has to be elucidated in further studies.

## Conclusion

In summary, the here presented chemotropic assay for the investigation of directed growth of filamentous fungi based on a 3D printed device advances the methods reported earlier. By using glass slides, it allows easy positioning under a standard upright microscope and enables the investigation of dynamic protein localization during chemotropic processes using fluorescence microscopy. The 3D printed combs further enable uniform signal gradient formation throughout different experiments, increasing the reproducibility of the assay. Due to these characteristics, the 3D printed device is ideally suited for the investigation of chemotropic growth responses like germling fusion in early colony development, host-pathogen and symbiotic interactions in the fungal community. Regarding pathogenic interactions, this method can further be applied for the identification of inhibitors of such and might help to improve food safety strategies and contribute to human health.

## Data Availability Statement

The raw data supporting the conclusions of this article will be made available by the authors, without undue reservation, to any qualified researcher.

## Author Contributions

CS performed experiments described in the manuscript, analyzed the data, and revised the manuscript. SP contributed to the experimental design and revised the manuscript. DN designed the study, contributed to experimental design, performed experiments described in the manuscript, analyzed the data, and wrote the manuscript. All authors contributed to the article and approved the submitted version.

## Conflict of Interest

The authors declare that the research was conducted in the absence of any commercial or financial relationships that could be construed as a potential conflict of interest.
