# Erratum: A 3D printed device for easy and reliable quantification of fungal chemotropic growth

**DOI:** 10.3389/fmicb.2023.1193234

**Published:** 2023-04-04

**Authors:** Frontiers Production Office

**Affiliations:** Frontiers Media SA, Lausanne, Switzerland

**Keywords:** chemotropism, 3D printed device, glucose, *Colletotrichum graminicola*, filamentous fungi

Due to a production error, in the **Methods** section, sub-section “Evaluation of Directed Growth Patterns,” the equations were incorrectly displayed. The correct equations are given below.


$$\begin{array}{l} \text{chemotropic index }\left[\text{\%}\right]=\left(\left\{\left(\frac{\text{number of attracted fungal tips}}{\text{sum of all fungal tips}}\right)*100\right\}-50\right)*2 \end{array}$$

$$\begin{array}{l} \text{chemotropic rate }\left[\text{\%}\right]=\left(\frac{\text{number of attracted fungal tips}}{\text{sum of all fungal tips}}\right)*100 \end{array}$$

The publisher apologizes for this mistake. The original article has been updated.

